# Ploidy Level and DNA Content of *Erianthus arundinaceus* as Determined by Flow Cytometry and the Association with Biological Characteristics

**DOI:** 10.1371/journal.pone.0151948

**Published:** 2016-03-24

**Authors:** Jiajun Yan, Jianbo Zhang, Kaiyan Sun, Dan Chang, Shiqie Bai, Yixin Shen, Linkai Huang, Jin Zhang, Yu Zhang, Yanhai Dong

**Affiliations:** 1 Sichuan Academy of Grassland Sciences, Chengdu, Sichuan, China; 2 Department of Grassland Science, Animal Science and Technology College, Nanjing Agricultural University, Nanjing, Jiangsu, China; 3 Department of Grassland Science, Animal Science and Technology College,Sichuan Agricultural University, Ya’an, Sichuan, China; University of Delhi, INDIA

## Abstract

*Erianthus arundinaceus* is not only an important germplasm resource for sugarcane breeding but also a potential bioenergy plant. Making clear the distribution of the chromosome ploidy of wild *E*. *arundinaceus* in china is the premise of the research and utilization of this species. Therefore, the objectives of this study were to determine the ploidy level and DNA content of the 55 *E*. *arundinaceus* accessions using flow cytometry and to identify the correlation between ploidy and phenotypic traits. Among the 55 accessions, four tetraploids and 51 hexaploids were identified. The four tetraploids originated from Mengma Yunnan, Shuangjiang Yunnan, Gaozhou Guangdong and Chengle Sichuan. The mean DNA content was 4.82 pg/2C for the tetraploid and 7.30 pg/2C for the hexaploid plants. The ploidy was negatively correlated with cellulose content and positively correlated (P<0.05) with plant height, stem diameter, leaf width, dry weight per plant, fresh weight per plant and hemicellulose content. However, ploidy was not correlated with leaf length, tiller number and the ratio of dry weight and fresh weight. This study will be useful for revealing the distribution of the ploidy of wild *E*. *arundinaceus* in Chin, traits markers analysis, and utilization of this species, such as cultivar improvement and sugarcane breeding in the future.

## Introduction

*Erianthus arundinaceus* is a perennial tall herb with dense clusters, and it is one of the most important wild species that are closely related to the genus of *Saccharum officinarum L*. [1]. *E*. *arundinaceus* plays an important role in breeding high-yield and high-sugar content sugarcane [2–4] due to its rich intraspecific variation, strong rooting and tillering ability, wide adaptability, and doughty resistance to abiotic and biotic stressors. Although it is difficult to hybridize *E*. *arundinaceus* with sugarcane, researchers have created some *Erianthus*-sugarcane hybrid materials [5–7]. Alternatively, *E*. *arundinaceus* is very suitable for use as a bioenergy plant [8] due to its high biomass yield, high photosynthetic efficiency and dry matter accumulation ability [9–11].

*E*. *arundinaceus* is naturally hexaploid. However, as breeders continue to improve the germplasm, the future use of *E*. *arundinaceus* could become complicated if the ploidy level of known germplasms is unclear. In addition, ploidy level is related to many desired characteristics, such as size, adaptation to stress, and intraspecific and interspecific hybridization ability [12, 13]. China has abundant *E*. *arundinaceus* resources in Xizang, Gansu, Henan, Shanxi, Zhejiang, Jiangxi, Hubei, Hunan, Sichuang, Yunnan, Fujian, Guangzhou, Guangxi, Guizhou, Hainan and Taiwan. Some chromosome number analyses have been reported and reflected three types of *E*. *arundinaceus* chromosome numbers: 2n = 20, 40 and 60. The 2n = 20 and 2n = 40 types are comparatively rare [14, 15]. Xiao [16] reported that the leaf width of *E*. *arundinaceus* was negatively and significantly correlated with ploidy level. Although the ploidy level of *E*. *arundinaceus* has been determined, the existing data are limited to a few cultivars.

DNA content reflects the entire and specific genetic information in all biological species [17]. Determination of the DNA content of a species not only provides important information for research on molecular genetics and cell genetics but also provides basic data for the research and genetic evolution of plant genomics. To date, the plant DNA C-values database has been established and includes 5150 species [18], including algae, mosses, ferns, gymnosperms, and angiosperms, among others [19, 20]. The DNA content in assorted plants has been determined [21]. However, little research on the DNA content of *E*. *arundinaceus* has been reported.

The traditional method for determining ploidy level is to count the number of chromosomes in a single plant cell, which has been used dependably for different species [22, 23]. However, this method is time-intensive and laborious, and the results may occasionally be inexact, especially when examining a large set of samples [24, 25]. Attempts have been made to distinguish ploidy level through morphological characteristics because plants differing in ploidy level may vary in growth habits. However, this method also has not always proven accurate. Flow cytometry has been widely used in the determination of ploidy level and DNA content and appears to be an efficient, pinpoint and powerful method [26, 27].

In our study, Sorghum ‘Bt * 623’ (*Sorghum bicolor* L.) was used as an internal standard [28], and 55 *E*. *arundinaceus* accessions were collected from different regions of China. Their ploidy level and DNA content were determined using flow cytometry. In addition, *E*. *arundinaceus* ‘Hainan92-105’ was used as external standard to verify the ploidy level [29, 30]. Therefore, the objectives of this study were as follows. First, ploidy level and DNA content were determined to provide basic data for the study of the genome. Second, cluster analysis of biological traits was performed as an important basis for the study of genetic differences among cultivars and the parental selection of cross breeding. Third, we assessed the correlation between ploidy level and DNA content and biological traits to quantify the impact of ploidy level.

## Materials and Methods

### Ethics Statement

This study was approved by Sichuan Academy of GrasslandScience; Department of Grassland Science, Animal Science and Technology College, Nanjing Agricultural University and epartment of Grassland Science, Animal Science and Technology College, Sichuan Agricultural University. No specific permissions were required for collecting *Erianthus arundinaceus* samples at the locations in China, because the research was funded by the National Natural Science Foundation of China, Ministry of Science and Technology and the earmarked fund for China Agriculture Research System of the People’s Republic of China, and the species is not an endangered or protected species.

### Plant material

The *E*. *arundinaceus* used in this study included 55 accessions collected from seven provinces, including Shaanxi, Sichuan, Guizhou, Guangxi, Yunnan, Guangdong, and Hainan (Sichuan Academy of Grassland Science, China). The internal standard material was Sorghum ‘Bt * 623’ (Jiangsu Academy of Agricultural Sciences), and the external standard material was *E*. *arundinaceus* ‘Hainan 92–105’ (Hainan Sugarcane Hybridization Station, Guangzhou Sugarcane Industry Research Institute, China). Each accession was planted in one area (5 meters in width, 8 meters in length), with one meter distance between each area and fifteen plants evenly distributed in each. In addition to weeding during the seedling stage, regular field management was performed. In the flowering stage, growth indicators were measured to assess the plants’ characteristics.

### Nuclear suspension and analysis of flow cytometry

Three plants were selected randomly for each accession, and a nuclear suspension was prepared according to the modified method of Arumuganathan and Doležel [31, 32]. First, to determine DNA content per nucleus, approximately 150 mg of fresh *E*. *arundinaceus* leaf tissue from each accession and 30 mg of sorghum leaf tissue (as an internal standard) were mixed and cut into 0.5- to 1.0-mm segments in a petri dish on ice containing 1.8 mL of solution (OttoI:OttoII = 2:1) (OttoI: 0.10 mol/L citric acid, 0.50% Tween 20; OttoII: 0.40 mol/L NaHPO_4_·12H_2_O). The excised leaf tissue was then filtered through 30-mm nylon mesh into a microcentrifuge tube and centrifuged at 5000 r/min, 4°C for 5 min. The supernatant was discarded, and the pellets were resuspended in 200 μL of OttoI, followed by the addition of 30 μL of RNase (Sigma-Aldrich Company, USA) and 50 μL of PI (propidium iodide, Sigma-Aldrich Company, USA). The resuspension was incubated at 4°C for 30 min. To determine ploidy level, approximately 130 mg of fresh *E*. *arundinaceus* leaf tissue of each accession was mixed in a petri dish on ice, containing 1.5 mL of solution followed by the same remaining steps. The nuclear suspension was analyzed immediately using an Accuri C6 flow cytometer (Becton Dickinson and America company, Franklin Lakeside. NJUSA), and 200,000 nuclei were collected.

The DNA content for each sample was proportional to the amount of PI intercalating in the double-stranded DNA. The DNA content per cell was also proportional to the fluorescence intensity of PI measured by the flow cytometer. The flow cytometer records the fluorescence intensity of cells in the G1 and G2 periods for the sample. The G1 period cells precede DNA synthesis (S), and the G2 period cells have accomplished DNA replication but have not split. Thus, the 2C DNA content was calculated based on the value of the fluorescence intensity of G1 peaks for both the internal standard and the sample. The ploidy level and DNA content of the unknown samples were calculated as follows:

$$\text{Ploidy Nuclear DNA content}=\frac{\text{Mean position of sample peak}}{\text{Mean position of standard peak}}*\text{Ploidy}/\text{DNA content of the standard}$$

### Measurement of traits

In the flowering stage, three individual plants for each accession were selected for measurement. The plant height and tiller number were measured from the soil surface to the top of the uppermost leaf blade. The leaf length and leaf width were measured at the reciprocal second leaves. The stem diameter was measured by tape. The stubble height used for measuring fresh weight (FW) and dry weight (DW) per plant was 10 cm. Physiological traits including cellulose, hemicellulose, lignin, and crude ash were determined by the method of VanSoest and Roberston.

$$\text{Water rate}=\text{1-}\frac{\text{Stand dry weight}}{\text{Stand fresh weight}}*\text{100}$$

### Data analysis

The images and data obtained by flow cytometry were analyzed using the Cytomics Expo32 software to obtain the value of the fluorescence intensity of the G1 peaks. The mean and standard error (SE) of the DNA content and traits for each tetraploid and hexaploid accession were analyzed using Microsoft Excel 2003. Correlations between the DNA content and traits of 55 accessions were determined using Statistical Product and Service Solution (IBM SPSS Statistics 20.0; StatSoft launch, 2011), which was also used for clustering analysis. In addition, a tree diagram was produced using system cluster analysis. SAS/MEANS was used to calculate means. SAS/CORR was used for calculation of correlation coefficients between ploidy level, DNA content and each agronomic and physiological traits in tetraploid and hexaploid of *E*. *arundinaceu*. SAS/GLM was used for ANOVA analysis for comparing ploidy level of each agronomic, physiological trait and DNA content (SAS Institute, 2011).

## Results

### *E*. *arundinaceus* ploidy level and DNA content

**Fig 1:**
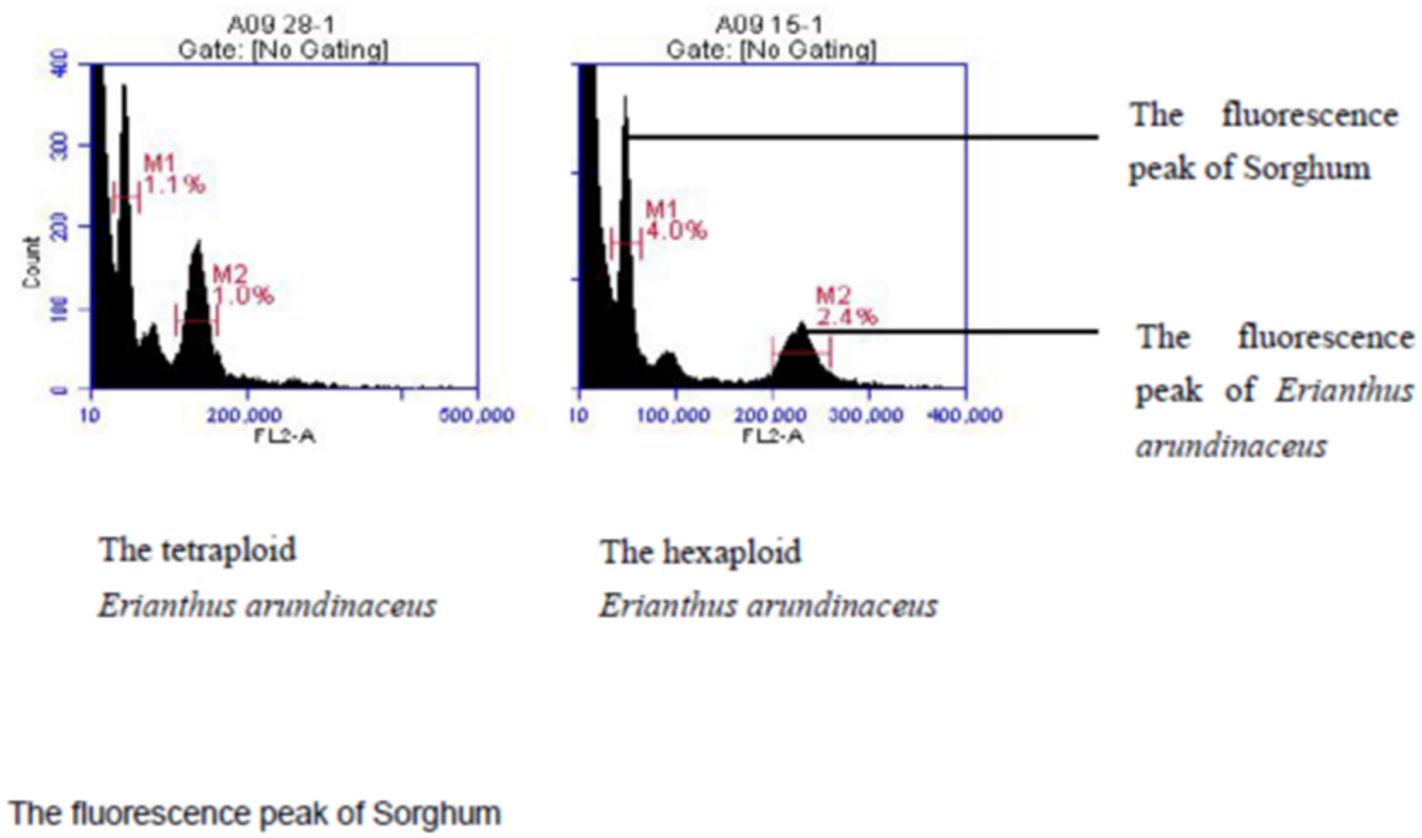
The fluorescence peak of tetraploid and hexaploid *Erianthus arundinaceus*.

The differences in DNA content between the tetraploid and hexaploid plants are indicated by the relative fluorescence intensity (Fig 1, S1 and S2 Datasets). Except for the meristem, which includes actively dividing cells, most of the cells were in the G1 period. Cells in the G2 period contained two-fold the amount of DNA compared with cells in G1. Among 55 accessions, four tetraploid and 51 hexaploid accessions were identified (Fig 1). The mean DNA content of the tetraploids and the hexaploids was 4.822 and 7.300 pg, respectively. Among the 51 hexaploids, the DNA content ranged from 6.981 to 8.391 pg for grasses from Sichuan province, 6.730 to 7.273 pg for grasses from Guizhou province, 6.980 to 7.416 pg for grasses from Guangdong province, 6.911 to 7.960 pg for grasses from Guangxi province, and 6.865 to 7.763 pg for grasses from Hainan province (Table 1). The tetraploids had DNA content ranging from 4.735 to 4.937 pg (Table 1). Two of these plants were from Yunnan province, and the other two plants were from the Sichuan and Guangdong provinces.

**Table 1:** The accession number(NO.), origin, altitude, latitude, longitude, ploidy level and mean DNA content of each accessions.

NO.	Origin	Altitude/m	Latitude/N	Longitude/E	DNA ±SE(pg)	Ploidy
E1	Hanzhong,Shanxi	801.0	32°32′22″	107°37′28″	7.407±0.026	Hexaploid
E2	Guangyuan,Sichuan	658.0	30°53′11″	105°48′45″	7.543±0.066	Hexaploid
E3	Shimian,Sichuan	1024.0	29°27′36″	102°11′29″	7.274±0.025	Hexaploid
E4	Xichang,Sichuan	1475.0	27°46′58″	102°12′42″	8.032±0.035	Hexaploid
E5	Panzhihua,Sichuan	962.0	26°37′23″	101°48′36″	7.274±0.060	Hexaploid
E6	Huili,Sichuan	1743.0	26°38′06″	102°15′03″	7.938±0.334	Hexaploid
E8	Ningnan,Sichuan	694.0	26°58′59″	102°48′23″	7.860±0.107	Hexaploid
E9	Heishui,Sichuan	2350.0	32°09′41″	103°02′49″	7.045±0.034	Hexaploid
E10	Hanyuan,Sichuan	891.0	29°26′40″	102°37′01″	7.066±0.098	Hexaploid
E11	Doujun,Guizhou	840.8	26°16′32″	107°29′58″	7.273±0.047	Hexaploid
E12	Dushan,Guizhou	943.8	25°45′32″	107°34′18″	7.330±0.060	Hexaploid
E13	Libo,Guizhou	546.3	25°27′22″	107°53′16″	7.075±0.039	Hexaploid
E14	Sandou,Guizhou	740.8	25°30′39″	107°31′53″	6.730±0.079	Hexaploid
E15	Congjiang,Guizhou	180.0	25°47′06″	109°03′50″	7.300±0.047	Hexaploid
E16	Rongjiang,Guizhou	235.0	25°56′20″	108°31′33″	7.232±0.027	Hexaploid
E17	Nandan,Guangxi	1127.0	25°06′08″	107°29′60″	7.128±0.041	Hexaploid
E18	Hechi,Guangxi	283.8	24°39′00″	107°51′49″	6.911±0.155	Hexaploid
E19	Duan,Guangxi	148.8	24°03′15″	108°02′46″	7.256±0.088	Hexaploid
E20	Wuzhou,Guangxi	39.0	23°29′27″	111°15′05″	7.084±0.097	Hexaploid
E21	Nanning,Guangxi	89.7	22°37′39″	108°23′02″	7.482±0.107	Hexaploid
E22	Shaoping,Guangxi	72.0	23°58′07″	111°06′01″	7.363±0.112	Hexaploid
E23	Zhongshan,Guangxi	157.6	24°27′26″	111°05′20″	7.960±0.044	Hexaploid
E24	Pingle,Guangxi	113.0	24°38′49″	110°38′22″	7.705±0.022	Hexaploid
E25	Guilin,Guangxi	166.0	25°18′43″	110°08′31″	7.454±0.048	Hexaploid
E26	Longsheng,Guangxi	226.0	25°48′11″	109°59′23″	7.485±0.108	Hexaploid
E27	Sanjiang,Guangxi	168.0	25°46′45″	109°38′47″	7.065±0.034	Hexaploid
E28	Mengma,Yunnan	531.0	30°23′54″	99°48′17″	4.937±0.068	Tetraploid
E29	Shuangjiang,Yunnan	670.0	29°32′42″	99°49′11″	4.815±0.070	Tetraploid
E30	Lancang,Yunnan	436.0	30°44′03″	99°48′30″	7.149±0.074	Hexaploid
E33	Suixi,Guangdong	43.0	21°33′26″	110°00′52″	7.416±0.152	Hexaploid
E35	Gaozhou,Guangdong	40.0	21°53′33″	110°49′46″	4.735±0.066	Tetraploid
E36	Xuwen,Guangdong	9.0	20°55′15″	110°03′32″	7.372±0.119	Hexaploid
E37	Xinyi,Guangdong	109.4	22°20′57″	110°54′44″	6.980±0.078	Hexaploid
E38	Hankou,Hannan	4.3	20°01′50″	110°19′44″	6.865±0.113	Hexaploid
E39	Dingan,Hannan	40.0	19°05′25″	110°10′47″	7.717±0.084	Hexaploid
E40	Tunchang,Hannan	140.0	19°12′10″	109°59′05″	7.763±0.126	Hexaploid
E41	Wuzhishan,Hannan	214.0	18°59′04″	109°33′56″	7.381±0.073	Hexaploid
E42	Baoting,Hannan	71.0	18°33′47″	109°37′20″	7.208±0.041	Hexaploid
E43	Dongfang,Hannan	-2.0	18°56′51″	108°41′59″	7.163±0.382	Hexaploid
E44	Sanya,Hannan	30.0	18°33′48″	109°37′20″	7.412±0.361	Hexaploid
E45	Sanyajiusuo,Hannnan	-32.0	18°25′47″	108°56′06″	7.489±0.203	Hexaploid
E46	Changjiang,Hannan	78.0	19°19′12″	108°58′32″	6.901±0.185	Hexaploid
E47	Danzhou,Hannan	19.6	19°43′06″	109°26′34″	7.148±0.183	Hexaploid
E48	Shuanghua,Sichuan	890.0	30°38′48″	104°01′18″	7.095±0.057	Hexaploid
E49	Suangliu,Sichuan	550.0	30°24′30″	103°54′00″	6.981±0.086	Hexaploid
E50	Jiajiang,Sichuan	426.0	29°47′19″	103°41′19″	4.800±0.185	Tetraploid
E51	Leshan,Sichuan	362.0	29°35′46″	103°46′09″	7.944±0.503	Hexaploid
E52	Meishan,Sichuan	408.0	30°21′04″	103°50′46″	7.896±0.036	Hexaploid
E53	Longquan,Sichuan	800.0	30°33′10″	104°19′34″	7.901±0.202	Hexaploid
E54	Yaan,Sichuan	584.0	29°58′55″	102°16′55″	6.965±0.156	Hexaploid
E55	Doujiangyan,Sichuan	700.0	31°02′09″	103°46′10″	7.222±0.096	Hexaploid
E56	Xinjin,Sichuan	460.0	30°25′28″	103°48′20″	8.391±0.104	Hexaploid
E57	Wenjiang,Sichuan	600.0	30°42′36″	103°46′30″	8.050±0.249	Hexaploid
E58	Suining,Sichuan	419.0	30°01′38″	105°32′29″	8.221±0.224	Hexaploid
E59	Jinjiangtang,Sichuan	625.0	30°40′51″	104°29′58″	8.162±0.106	Hexaploid

### Cluster analysis on plant biological characteristics

**Fig 2:**
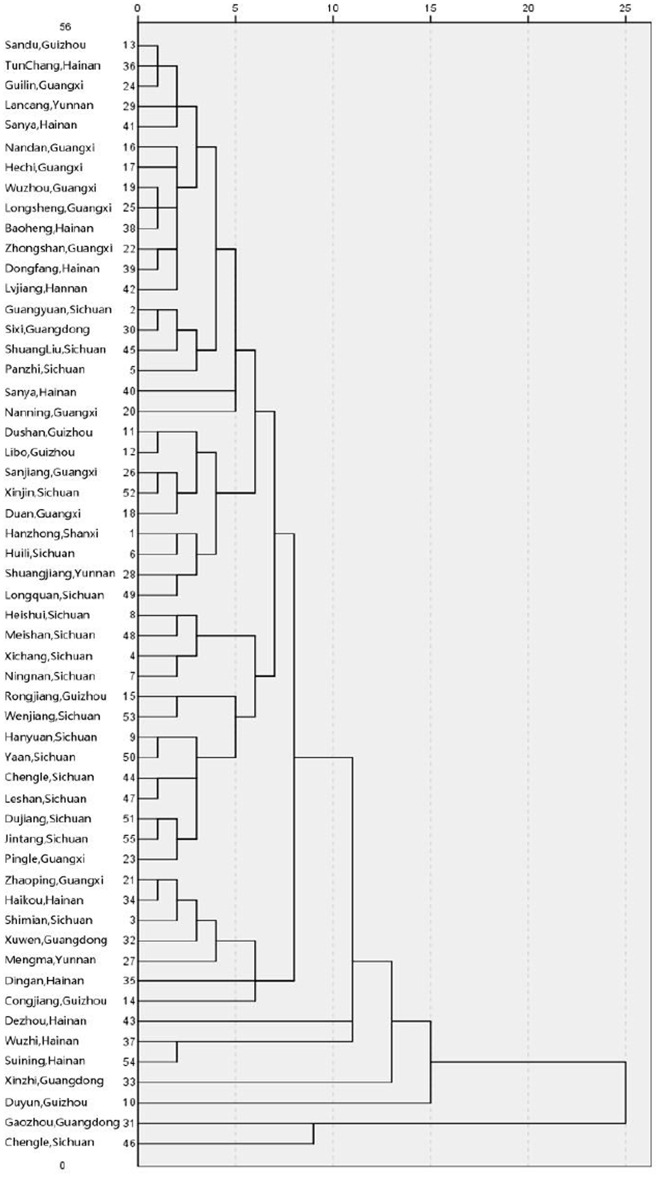
The tree diagram of cluster analysis based on plant traits.

Collection sites were used as variables in the cluster analysis. Based on the 12 traits of the 55 accessions (Fig 2), the between-groups linkage method was applied, and the results revealed that the 55 copies of *E*. *arundinaceus* from different provenances could be divided into 46 primary groups at a distance of 0.833 as a standard point. The clustering results revealed that the plants could be divided into six groups with a distance of 10.417 as a standard point. Of the 25 accessions in the first group, all were hexaploids, including nine accessions from Guangxi province, six from Hainan province, five from Sichuan province, three from Guizhou province and one each from Yunnan and Guangdong provinces. There were 25 accessions in the second group, two of which were tetraploid, including 14 accessions from Sichuan province, three from Hannan province, three from Guizhou province, a total of three from Yunnan and Guangxi provinces, and the last two from Shanxi and Guangdong provinces. The hexaploid accessions from Hannan, Guizhou and Guangdong provinces were considered as the third, fourth and fifth groups, respectively. The tetraploid accessions from Guangdong and Sichuan provinces were considered as the sixth group.

### Correlation between ploidy level and DNA content and agronomic traits

All agronomic traits exhibited continuous variation, which is typical of quantitative or polygenic inheritance. Plant height exhibited the largest variation. The second highest variation was noted in leaf length and tiller, and minimum variation was in the water rate (S3 Dataset). Specifically, the leaf width, tiller and stem diameter of hexaploids were approximately 1.5 times greater than noted in tetraploids, the other traits were 1.5 times lower, In addition the water rate of hexaploids was similar with tetraploids (Table 2). Moreover, the correlations between agronomic traits in the flowering stage and the ploidy level and DNA content were analyzed. The results revealed that the ploidy level was positively and significantly correlated (P<0.01) with plant height, stem diameter and leaf width, with correlation coefficients of 0.417, 0.493, and 0.404, respectively. The results also revealed that DNA content was positively and significantly correlated (P<0.01) with plant height and stem diameter, with correlation coefficients of 0.378 and 0.418, respectively. The ploidy level was also positively correlated (P<0.05) with leaf width, with a correlation coefficient of 0.312 (Table 2).

**Table 2:** The mean agronomic traits of tetraploid and hexaploid, their correlation with ploidy level and 2C DNA content in *E*. *arundinaceus*.

Traits	Tetraploid mean	Hexaploid mean	Ploidy	2C DNA
Plant height, cm	336.575±52.717	438.535±7.659	0.417**	0.378**
Stem diameter, cm	4.025±0.492	5.869±0.120	0.493**	0.418**
Leaf length, cm	158.200±15.832	177.676±2.973	0.228^NS^^†^	0.118^NS^
Leaf width, cm	2.125±0.423	3.494±0.115	0.404**	0.312*
Tiller number	67.000±18.000	104.000±7.000	0.183^NS^	0.109^NS^
SFW, kg	11.350±4.424	31.494±2.075	0.344*	0.226^NS^
SDW, kg	4.975±2.137	12.586±0.862	0.315*	0.191^NS^
Water rate, %	61.2±4.3	59.1±0.8	0.084^NS^	0.076^NS^

Note: SFW stand for fresh weight, SDW stand for dry weight,

^†^Non-significant at the 0.05 probability level,

**Significant at the 0.01 probability level,

*Significant at the 0.05 probability level.

### Correlation between ploidy level and DNA content and physiological traits

All physiological traits also exhibited a certain range of variation with different ploidy levels. Based on the results, hemicellulose content exhibited the largest variation. The second highest variation was noted in lignin content and cellulose content, and the least variation was in ash (S4 Dataset). The results indicate that except for hemicellulose content, hexaploid genotypes exhibited significantly reduced cellulose and lignin content compared with tetraploids, whereas ash contents were similar. However, in contrast to the agronomic traits, no multiple relationship was noted between hexaploid and tetraploid genotypes in terms of physiological traits (Table 3). In addition, the correlations between ploidy level and DNA content and physiological traits revealed that the ploidy level was positively and significantly correlated (P<0.01) with hemicellulose content, which was inversely correlated with cellulose content, and the correlation coefficients were 0.394 and 0.373, respectively. Moreover, the ploidy level exhibited no significant correlation with lignin content or ash content (Table 3). The results also revealed a negative relationship (P<0.05) between cellulose content and DNA content. In contrast, the hemicellulose content was positively correlated (P<0.05) with DNA content. These correlation coefficients were 0.275 and 0.327, respectively. No correlations were observed with other traits (Table 3).

**Table 3:** The mean physiological traits of tetraploid and hexaploid, their correlation with ploidy level and 2C DNA content in *E*. *arundinaceus*.

Traits	Tetraploid mean	Hexaploid mean	Ploidy	2C DNA
Cellulose, %	41.408±0.648	38.867±0.222	-0.394**	-0.275**
Hemicellulose, %	26.539±0.826	29.127±0.239	0.373**	0.327**
Lignin, %	12.574±1.059	11.311±0.207	-0.214^NS^^†^	-0.076^NS^
Ash, %	5.420±0.511	5.415±0.123	-0.002^NS^	-0.087^NS^

Note:

^†^Non-significant at the 0.05 probability level,

**Significant at the 0.01 probability level.

### ANOVA analysis for comparing accessions of different ploidy based on agronomic, physiological traits and DNA content

In this study ANOVA analysis was use to compare ploidy level (tetraploid and hexaploid) based on agronomic, physiological traits and DNA content, and also was used to compare the ploidy tetraploid and hexaploid accessions of each agronomic, physiological trait and DNA content, respectively (Table 4). The difference of plant heigh, stem diameter, leaf width, cellulose, hemicellulose and DNA content between tetraploid and hexaploid were significant, and other traits were not significant. In 4 accessions of tetraploid, the difference of traits were significant except the water rate and DNA content. In 51 accessions of hexaploid, the difference of all traits were significant.

**Table 4:** ANOVA analysis for comparing ploidy level (tetraploid and hexaploid), accessions of tetraploid, and accessions of hexaploid based on agronomic, physiological traits and DNA content, respectively.

Traits	Plant height cm	Stem diameter cm	Leaf length cm	Leaf width cm	Tiller number	Water rate %	Cellulose%	Hemicellulose %	Lignin %	Ash %	DNA content %
Ploidy	11.17**	16.43***	2.92^NS^^†^	10.05**	1.84^NS^	0.37^NS^	9.73**	8.59**	2.55^NS^	0.00^NS^	167.04****
Accessions (tetraploid)	160.86****	49.03****	22.33****	50.77****	32.18****	2.34^NS^	6.54*	12.56**	40.86****	10.88**	0.59^NS^^†^
Accessions (hexaploid)	17.39****	13.86****	10.46****	26.16****	46.76****	4.82^NS^	3.22****	1.87**	10.31****	17.69****	6.74****

Note:

^†^Non-significant at the 0.05 probability level;

*Significant at the 0.05 probability level;

**Significant at the 0.01 probability level;

***Significant at the 0.001 probability level;

****Significant at the 0.0001 probability level.

## Discussion

### *E*. *arundinaceus* ploidy level and DNA content

This study is the first to determine the ploidy and DNA content levels of *E*. *arundinaceus* by flow cytometry. The use of flow cytometry saved time and resources. The protocol developed in this research could be used as a reference for researching *E*. *arundinaceus* by flow cytometry in future work. First, based on the complexity of plant cell nuclei, an appropriate buffer was critical for obtaining an ideal nuclear suspension. Second, the sampling of new leaves from young canes was important because older leaves can produce fewer intact nuclei, resulting in reduced fluorescence. Third, the double blade should be kept sharp to reduce mechanical damage to the nuclei.

Because the G1 peak of Sorghum was close to the peaks of *E*. *arundinaceus*, but not overlapping, Sorghum was selected from the list of plants recommended as excellent DNA content standards [33]. This choice is also reasonable for consistency with previous research examining the DNA content in gramineous plants (Fig 2). Both the internal and external standard methods were used to determine ploidy level. The results indicate that the fluorescence peak measured by the internal standard method was more accurate. This result may be because the two types of plant simultaneously underwent dissociation and staining in the internal standard method, which substantially reduced the artificial error. Therefore, the internal standard method is recommended for the determination of *E*. *arundinaceus* ploidy levels. It is also worth mentioning that leaves were used as the experimental materials and may exhibit endogenous polyploidy. However, the cause of endogenous polyploidy and its biological function require further research.

Only four tetraploids were identified among 55 accessions (Table 1), indicating that most *E*. *arundinaceus* is hexaploid in nature. The results were similar to the previous reports. [14, 34, 35]. Although only 7.2% of the selected accessions were tetraploid, their presence could complicate research results when choosing and using natural populations as well as providing important information for the breeding of sugarcane.The gene controlling biomass related traits like stem diameter and plant height of tetraploid *E*. *arundinaceus* could be much easier to transferred into sugarcane than hexaploid *E*. *arundinaceus* for its relative smaller genomes.

In this study, all agronomic and physiological traits of tetraploids *E*. *arundinaceus* got from 4 tetraploids *E*. *arundinaceus* accessions, the number of accessions was much less than hexaploid *E*. *arundinaceus* accessions, and unfortunately, it is very difficult for us to get more wild tetraploids *E*. *arundinaceus* accessions. To evaluate whether 4 tetraploids *E*. *arundinaceus* accessions were sufficient to capture the potential variability, the ANOVA analysis for comparing 4 accessions of tetraploid was studied, the difference of traits were significant except the water rate and DNA content, so 4 tetraploid accessions cloud capture a certain potential variability, but for capturing enough potential variability, it is necessary to collect more hexaploid *E*. *arundinaceus* accessions.

The mean DNA content of the tetraploids and the hexaploids was 4.82 and 7.30 pg, respectively. However, it is worth mentioning that the use of different sampling procedures and internal standards might cause slight differences between individual studies. The DNA content of the hexaploids was not exactly 1.5-fold increased compared with the tetraploids. Wang et al. [36] found similar results in 200 perennial ryegrasses, where the DNA content of tetraploids was not exactly two-fold increased compared with diploids. Genome differences among species could be the reason for this phenomenon. The grasses from Sichuan province exhibited the largest variation in DNA content, whereas grasses from Guangdong province exhibited the smallest variation. The large sample size might contribute to the relatively large variation in DNA content in the samples. Above all, although the study is helpful for sugarcane breeding, *E*. *arundinaceus* trait marker analysis, cultivar improvement, genome sequencing, genomic library construction and genomics research, the measurement of larger sample sizes is recommended to provide a genomic background for further *E*. *arundinaceus* breeding programs.

### Cluster analysis of plant traits

Different *E*. *arundinaceus* resources were applied to cluster analysis, which could provide an important basis for the study of genetic differences among cultivars and the parental selection of cross breeding. It is feasible to use the method of clustering analysis because the basic data were obtained from seven provinces, thus ensuring genotype differences. The results of cluster analysis were intuitive and objective [3, 4]. In this study, 55 accessions were polymerized depending on 12 traits. Unlike most reports [10], these results indicate that the groups did not correspond simply to each collection site; however, certain regionalisms still existed. Although a certain correlation was noted between the characteristics of different accessions and their attribution, the differences were not identical, which may relate to differences in habitat conditions within the same province. Therefore, both the region and the characteristics should be considered when selecting parents.

The cluster analysis results revealed rich diversity in the genetic variation of the main characteristics of *E*. *arundinaceus* [10], which might contain important gene resources to be discovered for sugarcane breeding. According to the comprehensive comparison of the 12 main characteristics of the 55 materials, an important conclusion was also drawn that ideal parent materials could be chosen from the first group, whose stem diameter, plant height and cellulose content were better, because better stem diameter and plant height could help increase sugarcane yield, and more cellulose content could help the processing of sugarcane. However, parental selection should not be kept within the group because the germplasm resources of the group exhibited a smaller genetic distance between each other. In contrast, the germplasm resources belonging to the other groups exhibited greater differences.

Another trend in the evolution is also observed. Although the tetraploid and hexaploid materials were not simply and directly clustered into two categories, it was obvious that the results still revealed a certain polymerizability. Therefore, it was confirmed that the ploidy level was correlated with overall traits. Above all, the cluster analysis of *E*. *arundinaceus* between resistance and other important traits should be further studied.

### Correlation between traits and ploidy level and DNA content

The main target of assessing the correlation between ploidy and traits was to quantify the impact of ploidy level. Kamemoto et al. [37] believed that polyploidy would show larger plant type, leaf and petal size. Sari et al. [38] found that the flowers of tetraploid watermelons were larger and more compact compared with diploids. Moreover, when the ploidy level changed from tetraploid to diploid, the size of flowers, pollen grains and fruit decreased. These results were consistent with the finding that the mean of each hexaploid *E*. *arundinaceus* trait was greater compared with tetraploid accessions. Another discovery of this study was that ploidy level was negatively correlated with cellulose content and positively correlated with plant height, stem diameter, leaf width, water rate and hemicellulose content. Therefore, if the breeding target of *E*. *arundinaceus* is a large plant, hexaploid varieties should be bred. If the breeding target is a plant with high cellulose content, tetraploid varieties should be choses.

Bonos et al. [39] found that only flag leaf length was positively correlated with DNA content in six *Agrostis* species, and no other morphological measurements were associated with DNA content. In addition, Wang et al. [36] reported that plant height was positively correlated with DNA content, but leaf width at the seedling stage in perennial ryegrass was not. Their research results were not analogous to ours. While agronomic factors are an important element in bioenergy crop production, physiological traits should also be considered [40]. One study indicated that the diploid perennial ryegrasses exhibited significantly increased natural detergent fiber (NDF) and acid detergent fiber (ADF) values compared with their tetraploid derivatives, whereas the situation was completely reversed for digestible organic matter (DOM) [41]. Some research has suggested that triploid and tetraploid genotypes exhibit significantly reduced lignin content compared with diploid genotypes at harvest, but cellulose content does not vary significantly with ploidy [42]. The data from these studies are also not consistent with ours. These results revealed that the degree of correlation in different plants was not completely consistent. In addition to the underlying genetic control, plant traits may be significantly impacted by environmental factors and plant growth habits, adding to the complexity of these highly polygenic traits and warranting further research.

It is worth mentioning that the results also indicate that not all individual characteristics of hexaploids were increased or decreased compared with tetraploid *E*. *arundinaceus*, suggesting that the identification of ploidy level solely through morphological measurements is not a reliably accurate method. In addition, the results of this study indicate that water rate was positively correlated (P<0.05) with the ploidy level but were not correlated with DNA content. Moreover, the correlation coefficients of traits and ploidy were not equal to the correlation coefficients of traits and DNA content, most likely due to differences among groups or the interspecific genetic basis of *E*. *arundinaceus*. These results regarding the relationship between ploidy and traits could provide further insight into yield and biomass quality improvements in *E*. *arundinaceus*. In addition, the correlations between ploidy level and DNA content and resistance to disease, pest, drought, flood and barrenness need to be studied.

In summary, flow cytometry can be used to determine ploidy level and DNA content in *E*. *arundinaceus*. The ploidy levels of 55 Chinese *E*. *arundinaceus* accessions were successfully identified using flow cytometry. Among them, four tetraploids and 55 hexaploids were identified. The average DNA content was 4.82 pg/2C for the tetraploids and 7.30 pg/2C for the hexaploids. On one hand, the results of the cluster analysis indicated that ploidy level was correlated with its overall traits. On the other hand, the specific correlation analysis found that the ploidy level was negatively correlated with cellulose content and positively correlated with plant height, stem diameter, leaf width and hemicellulose content. Determining *E*. *arundinaceus’* ploidy level is conductive to establishing the population structure necessary for marker trait association analysis and natural tetraploid mapping populations to analyze genetic diversity. In addition, the four tetraploid accessions may also be potential resources for the cultivar improvement and breeding of sugarcane.

## Supporting Information

S1 Dataset The fluorescence value of internal standard material. (XLS)

S2 Dataset The fluorescence value of external standard material. (XLS)

S3 Dataset Agronomic traits of 5 replications. (XLS)

S4 Dataset Physiological traits of 3 replications. (XLS)
